# Impact of maternal diabetes and obesity on fetal cardiac functions

**DOI:** 10.1186/s43044-020-00077-x

**Published:** 2020-07-31

**Authors:** Suzan Bayoumy, Marwa Habib, Randa Abdelmageed

**Affiliations:** Cardiology Department, Faculty of Medicine, Tanta University, Tanta, Egypt

**Keywords:** Obesity, Diabetes, Fetal echocardiography, Cardiac functions

## Abstract

**Background:**

In several developing industrial countries, the incidence of obesity among populations is spreading quickly and dramatically; also, the frequency of maternal obesity is in continuous elevation, which represents a considerable public health problem. Maternal hyperglycemia is a common gestational risk factor for the fetus. Several studies proposed that maternal DM and obesity lead to intrauterine impacts which induce changes in the fetal myocardium, and the pre-pregnancy obesity and diabetes are accompanied with development of cardiovascular alterations in the offspring and subsequent pathological changes in their early life. The aim of this study is to assess the cardiac function in fetuses of obese pregnant women (FOW) and fetuses of diabetic women (FDW) in comparison with fetuses of normal pregnant women (FNW) using tissue Doppler imaging.

**Results:**

There was impairment in systolic and diastolic cardiac function in both fetuses of obese and diabetic women with decreased global longitudinal strain tissue Doppler velocities at 30 weeks of gestation compared to fetuses of normal women.

**Conclusion:**

Imaging of the fetus of pregnant women by Echo Doppler at about 30 weeks of gestations showed a reduced cardiac function of fetuses of obese and diabetic women matched with fetuses of normal BMI women. Our finding proposed that early subclinical alterations in the fetal cardiac output can arise from maternal obesity alone. This explains the predilection of children of obese mothers at advanced ages to cardiovascular disorder.

## Background

The main public health problems all over the world are overweight (obesity), type 2 diabetes, and cardiovascular disorder. These communal disorders have a great effect on morbidity and mortality in the overall mature people [1]. Maternal obesity and diabetes seem to have persistent effects on offspring outcome, which increases the risk of fetal and neonatal complications including prematurity, stillbirth, macrosomia, and congenital anomalies [2]. Maternal obesity may also predispose the offspring to hypertension, type 2 diabetes, dyslipidemia, and heart disease as well. Studies have also demonstrated that children of obese women are at greater risk for congenital heart defects and myocardial hypertrophy [3].

Obesity is a chronic low-grade inflammatory disease. The result of this inflammation is endothelial dysfunction, hypertension, insulin resistance, and cardiac dysfunction; fibrosis occurs in response to inflammation. Some inflammatory markers such as C-reactive protein and transforming growth factor (TGF)-β have been shown to enhance collagen accumulation in fetal myocardium; most likely, this induces fibrosis in fetal myocardium and impairs systolic and diastolic function and alter cardiac morphometry which leads to increase in wall thickness and hypertrophy [4].

These changes in cardiac function in fetuses of obese mothers (subclinical cardiac changes) could be measured by echocardiographic techniques such as speckle tracking and tissue Doppler [3].

Maternal diabetes is associated with increased risk of fetal morbidity, stillbirth, and neonatal morbidity and mortality; hyperglycemia and hyperinsulinemia and high content of insulin receptors lead to increase in growth factors, hyperplasia, and hypertrophy of the myocardium of the fetal heart; diastolic dysfunction is the earliest changes preceding systolic dysfunction [5].

The assessment of fetal myocardial function is performed by professionals in fetal cardiology. Fetal echocardiography has great advances in recent years and is characterized by the following: an easily accessible, non-invasive method and can be applied during gestation to explore the cardiac anatomy and function of the fetus accurately.

Moreover, evaluation of the myocardial performances depends in majority of investigations on conventional Doppler US, which demonstrates global cardiac function. Fetal cardiac function can be evaluated by multiple echocardiographic methods. Tissue Doppler imaging (TDI) which is used for myocardial function evaluation is characterized by the possibility of direct estimation of regional myocardial velocity and can detect earlier changes in myocardial function; in addition, it offers more sensitive imaging technique [6].

Also, for evaluating myocardial deformation, another technique is used which is an angle-independent method “two-dimensional speckle-tracking echocardiography (2D-STE).” One advantage of this method is that tracking occurs in two dimensions along the direction of the wall and not along the ultrasound beam: therefore, STE is considered as angle independent [7].

For estimation of preclinical myocardial dysfunction in children and adults, both Doppler parameters, strain and strain rate by 2D-STE, are used for this purpose [8, 9]. Using speckle tracking during gestation for evaluation of fetal cardiac condition is practicable but has a number of tasks along intrauterine life [10]. In women suffering from diabetes mellitus, fetal echocardiography is used for detection of hypertrophic cardiomyopathy and congenital heart disease which is considered part of the general fetal scanning examination.

We postulated that obesity and maternal diabetes had an impact on fetal cardiac outputs; therefore, the purpose of this work was to assess the cardiac functions in the fetal heart of obese women (FOW) and fetuses of diabetic women (FDW) in comparison with fetuses of normal pregnant women (FNW).

## Methods

### Study participants

This was a prospective study which comprises eighty pregnant women who were examined by fetal echocardiography during the period from 2016 to 2018 from 25 normal pregnant women (group A), 50 obese pregnant women (group B), and 25 pregnant women with pregestational diabetes (group C).

Group B (obese women) is composed of those with BMI 30 kg/m^2^. BMI is body mass index.

The inclusion criteria of participating women were the following: BMI was ≤ 25 kg/m^2^ in normal pregnant women and ≥ 30 kg/m^2^ in obese pregnant women estimated before conception; other grades of obesity were excluded, and also BMI > 40% were excluded. Women who had fetal cardiac abnormalities and multiple pregnancies were not involved in the study. Patients with hypertensive heart disease were excluded from the study; all patients were normotensive.

Patients with diabetes were controlled during pregnancy without complications of diabetes. HbA1c was about 6.5 in the diabetic group; HbA1c was done for obese and normal patients for exclusion of diabetes at the beginning of the study, and it was 5.5. The examinations were done at approximately 30 ± 2 weeks of gestation. An illustrated written consent was taken from all participants. The approval letter on the proposed protocol of this investigation study was obtained from the ethical committee of the faculty of the participating authors.

### Echocardiography

The ultrasound which is used in the current study is the most common one of US which are used for assessment of the fetal heart. Gel substance was spread on the abdomen of the mother; then, the probe of ultrasound is smoothly positioned on the mother’s abdomen, and images were taken during scanning using vivid 9 M5S probe. The examination was done at approximately 30 ± 2 weeks of gestation. The diagnosis by US was easy to perform, not painful, and caused no injury to the fetus. The examination persisted for an average of 30–45 min according to the position of the heart of the fetus. EchoPAC PC version BT 12 was used for the analysis of TDI recordings during offline. All obtained data constitute the mean of three successive recordings of each evaluation, which were saved on CD/DVD. All fetuses were studied. A full diagnosis was performed for the anatomical structure of the heart of fetus, via taking five transverse images along the thorax and the fetal abdomen.

### Annular pulsed wave tissue Doppler velocity (TDV)

Color-coded tissue Doppler US was adjusted at high frame rates and analyzed offline, by employing an area of concern at the basal level of the left segment adjacent to the mitral ring and the right segment neighboring to the tricuspid rings.

Platform-specific software then generates velocity view over the cardiac cycle, including E’ and A’. The velocity S’ was considered the peak of systolic velocity, lacking over gaining the Doppler envelop. For detecting tricuspid annular plane systolic excursions (TAPSE), M-mode cursor was placed at a right angle to the atrio-ventricular junction, noticeable by the valve rings at the tricuspid valve.

At peak systole from a standard apical 4-chamber window, the quantity of longitudinal movement of the annulus was estimated by using 2D-echocardiography (measured in millimeter). Global longitudinal strain (GLS) via using speckle-tracking echocardiography (STE) and longitudinal two-dimensional (2D) image was evaluated, whereas the digital loops were attained from the three apical images, where the good-quality 2D scans of cardiac cycle were chosen.

In order to describe the area of importance and outline the internal border of the myocardium, EchoPAC, GE (a semi-automatic method) was used offline. Manually adjusted segments that failed to be tracked and the segments that failed to be tracked were excluded. Area of importance was adjusted manually if it is essential for measuring the mean of the thickness of the myocardium. The software algorithm measured automatically 2D strain at each frame along the cardiac cycle as a curve for each apical view.

#### Statistical analysis

Statistical presentation and analysis of the current investigation were performed by SPSS V17 that involve the mean ± standard deviation and Student *t* test. To compare the three groups in quantitative data, unpaired Student *t* test was applied. Descriptive data were expressed for normally distributed data and median, while for non-normally distributed data, mean USD and interquartile range was used. *P* value less than 0.05 was considered a significant difference. An interaction between the three groups (obese, diabetic, and normal) was involved in the statistical design to evaluate whether potential alteration in the data estimated varied among the fetus of normal women, obese women, and diabetic women. The official approval on the protocol of the current proposed study was dependent by the local ethical committee, and all pregnant women participating in this study wrote consent before this study.

## Results

This study included 80 pregnant women: 25 normal gestational women, 30 obese gestational women, and 25 pregnant women with pregestational diabetes.

The mothers’ age ranged from 20 to 37 years examined at 30 ± 2 weeks of the gestational stage. As regards parity and age, there were no significant differences among the studied groups. All of the pregnant women participating in the study were non-smokers and non-hypertensive. All fetuses were assessed by full 2D echocardiographic assessment, as well as tissue Doppler imaging with special attention to ventricular functions comparing fetuses of normal women (FNW) group A with fetuses of obese women (FOW) group B and fetuses of diabetic women (FDW) group C.

The percentage of each group relative to the total number of cases was group A represents 31.25% of the total number of patients, group B represents 37.5% of the total number of patients, and group C represents 31.25% of the total number of patients.

The main finding of the present study was that fetuses of obese women had significantly impaired fetal myocardial function measured by global longitudinal strain in fetuses of obese and diabetic mothers compared to normal mothers; tissue Doppler velocities were significantly lower among the fetuses of obese and diabetic mothers compared to normal pregnancy.

In this study, there was a significant increase in interventricular septal thickness and thickening of the left ventricular free wall which was similar to the findings reported in fetuses of diabetic mothers.

There is a table and reference for normal values of tissue Doppler velocities in the fetus according to gestational age [11].

In Table 1, left annular velocity was measured by color-coded tissue Doppler. Early diastolic (E’) and late diastolic (A’) peak pulse wave and systolic (S’) tissue Doppler velocities were determined from the LV annuli of the apical four chambers. Left annular E wave velocity was 4.878 ± 0.245 cm/s in group A, 4.398 ± 0.320 cm/s in group B, and 4.567 ± 0.425 cm/s in group C. The *P* value was < 0.001 between groups A and B, was 0.005 between groups A and C, and was 0.159 between groups B and C. Left annular A wave velocity was 8.067 ± 0.256 cm/s in group A, 6.297 ± 0.343 cm/s in group B, and 6.195 ± 0.420 cm/s in group C. The *P* value was < 0.001 between groups A and B, was 0.005 between groups A and C, and was 0.525 between groups B and C. Left annular A wave velocity was 5.600 ± 0.206 cm/s in group A, 4.437 ± 0.246 cm/s in group B, and 4.568 ± 0.318 cm/s in group C.

**Table 1:**
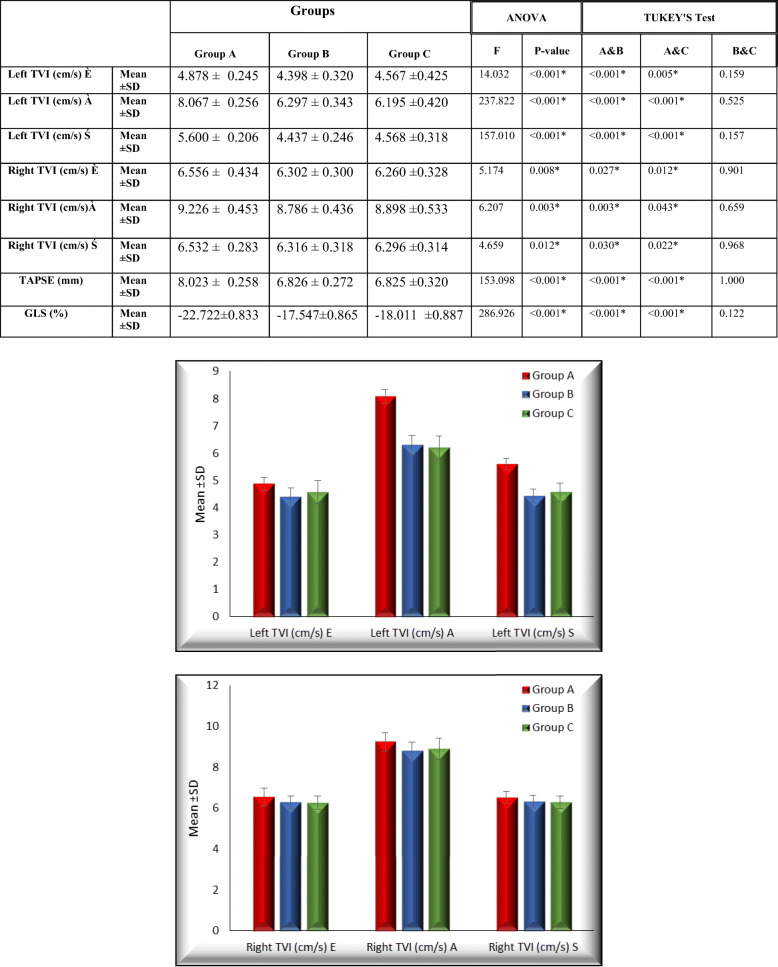
There is a significant difference between groups B, C, and A

The *P* value was < 0.001 between groups A and B, was 0.005 between groups A and C, and was 0.157 between groups B and C, which represent a significant variation in the left annular velocity between fetuses of diabetic and obese women matched with fetuses of normal women while there is non-significant variation recorded between fetuses of obese and diabetic women. Color-coded tissue Doppler was used for measuring the right annular velocity.

Right annular E wave velocity was 6.556 ± 0.434 cm/s in group A, 6.302 ± 0.300 cm/s in group B, and 6.260 ± 0.328 cm/s in group C. The *P* value was 0.027 between groups A and B, was 0.012 between groups A and C, and was 0.901 between groups B and C. Right annular A wave velocity was 9.226 ± 0.453 cm/s in group A, 8.786 ± 0.436 cm/s in group B, and 8.898 ± 0.533 cm/s in group C. *P* value was 0.003 between groups A and B, was 0.043 between groups A and C, and was 0.659 between groups B and C. Right annular A wave velocity was 6.532 ± 0.283 cm/s in group A, 6.316 ± 0.318 cm/s in group B, and 6.296 ± 0.314 cm/s in group C. *P* value was 0.030 among groups A and B groups, was 0.022 among groups A and C, and was 0.968 among groups B and C groups, which represents a significant variation of right annular velocity among foeti of diabetic and obese women matched with foeti of normal women, whereas a non-significant difference was recorded among fetuses of obese and the fetuses of DM women.

Tricuspid annular-plane systolic excursion (TAPSE) was measured by M-mode from an apical four-chamber view. TAPSE was 8.023 ± 0.258 mm in group A, 6.826 ± 0.272 mm in group B, and 6.825 ± 0.320 in group C. The *P* level was < 0.001 between groups A and B, < 0.001 between groups A and C, and was 1.000 among groups B and C, which shows a significant variation in TAPSE among fetuses of diabetic and obese women matched with fetuses of normal women, while a non-significant difference was observed among fetuses of obese and fetuses of diabetic women.

**Fig. 1:**
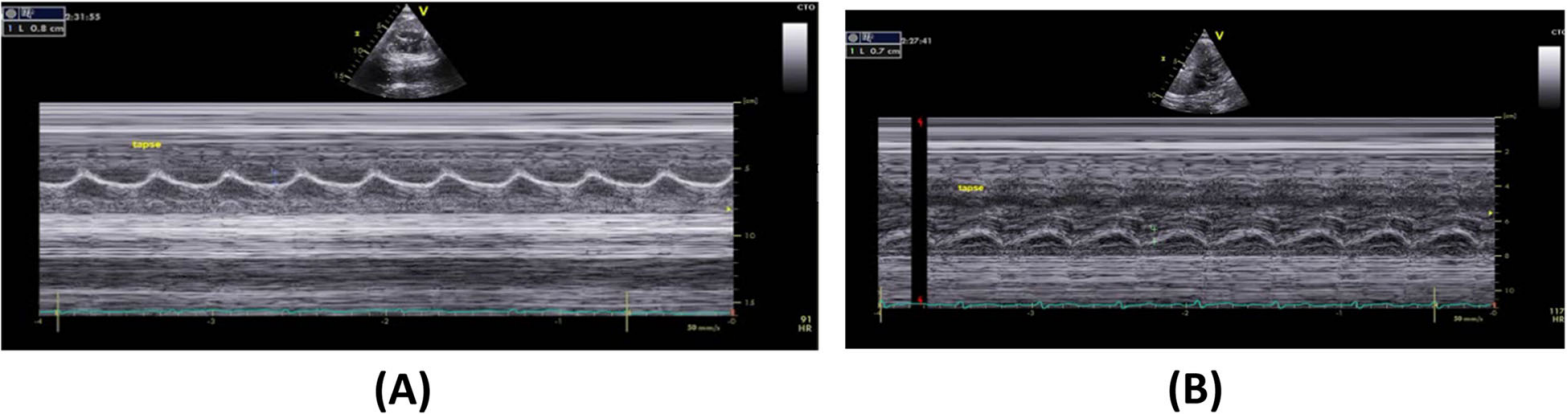
Tissue Doppler septal annular plane systolic excursion (SAPSE) in the fetus of **a** diabetic mother and **b** obese mother

**Fig. 2:**
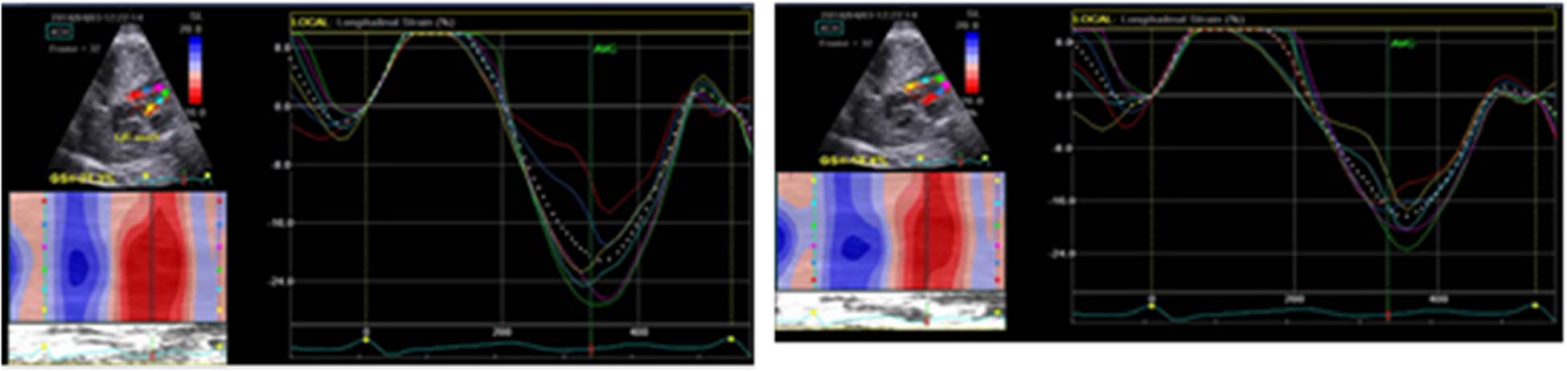
Global longitudinal left ventricular strain in the fetus of a normal weight mother and obese mother

Longitudinal left ventricular two-dimensional (2D) strain by speckle-tracking echocardiography (STE) was carried out. The left ventricular global longitudinal strain was − 22.722 ± 0.833% in group A, − 17.547 ± 0.865% in group B, and − 18.011 ± 0.887 in group C. *P* value was < 0.001 among groups A and B, < 0.001 among groups A and C, and 0.122 among groups B and C. There is a significant difference between groups B, C, and A (Figs. 1 and 2).

## Discussion

In order to invetigate the impact of maternal obesity and diabetes on fetal cardiac function both tissue Doppler imaging and speckle tracking are used.

The foremost result of the current investigation was that fetuses of diabetic and obese women had reduced significantly fetal myocardial function estimated by global longitudinal strain, nearly at 30 ± 2 weeks of pregnancy age. Global longitudinal strain of the left ventricle was diminished in fetuses of diabetic and obese women compared to fetuses of normal women.

Also, there was a decrease in the tricuspid annular-plane systolic excursion in fetuses of obese and diabetic women compared to fetuses of normal women, and annular tissue Doppler velocity was significantly lower among the fetuses of obese and diabetic women, matched with fetuses of normal women. This agrees with the finding of Ece et al. [12] in their study for assessments of the influence of pre-pregnancy maternal obesity on fetal cardiac performance.

Using standard echocardiography to assess fetal cardiac performance (MAPSE and TAPSE and mitral and tricuspid E, A, and E/A ratio) demonstrated that all fetuses seem to be apparently healthy. With respect to indices of ventricular systolic function, non-significant variations were recorded in this study, but TDI velocities of IVS, LV lateral wall, and RV free wall exhibited reduced diastolic output in fetuses of obese women.

In the current study, we found significant variations in the annular TDV among the two groups, but the TAPSE showed decreased data in the current study. The presence of diastolic myocardial failure in fetuses of women suffering from DM was reported before in many studies. In the meantime, the causal of biological mechanisms for diabetes and obesity are suggested to be a multifactorial.

Comparable elucidations are probable concerning elevated cardiovascular risk [13]. Vrachnis et al. [13] proposed that maternal obesity and/or diabetes convene a fetal hyperglycemia/hyperinsulinemia, which consequently leads to stimulation of insulin signaling pathway dysfunction of fetal adipocyte cell, and proinflammatory mediators of the myocardium. In another study carried by Turan et al. [14], there was a significant variation in the first trimester diastolic myocardial function of fetuses of poorly controlled diabetic mothers compared to non-diabetic controls. Also, Nyrnes et al. [15] in their study assessed cardiac performance in the offsprings of obese women and the offsprings of normal BMI women and had shown impaired systolic and diastolic cardiac function with diminished global longitudinal strain in the left and right ventricle, strain rate, tissue Doppler velocities and MAPSE at birth, and still at 6 ± 8 weeks after delivery compared to newborns of normal weight mothers.

These studies’ data were in agreement with the current study except for MAPSE which did not decrease in the present study, but the TAPSE was significantly reduced. The original biological mechanism for this variation was indistinct, but the reason for such damage may be the inflammatory response in the fetal myocardium in obese and diabetic women. Also, the increased pulmonary vascular resistance in utero may be the possible cause. The alteration in cardiac output in addition to pulmonary and systemic afterload, preload, and stress occurred after birth.

The changes in cardiac output as well as systemic and pulmonary preload, afterload, and resistance occurred after birth. The findings of the current study of fetuses of obese and diabetic women were significantly impaired fetal cardiac functions, and these results were similar to the findings reported by Al-Biltagi et al. [16] in neonates of diabetic mothers who evaluated the myocardial alterations in fetuses of diabetic women both with prepregnancy or pregnancy of diabetic women and compared this to the control group by using tissue Doppler scanning and two-dimensional speckle tracking scanning. This study showed that a significant alteration of both diastolic and systolic outputs and global strain determined by both tissue Doppler imaging and conventional echocardiography in those neonates of pregnant and pregestational diabetes matched with the control group.

## Conclusions

Cardiac function in fetuses of obese and diabetic pregnant women was diminished compared to fetuses of normal pregnant women at about 30 weeks of gestation during examination by tissue Doppler imaging (annular velocities and global left ventricular strain). Tissue Doppler imaging allowed non-invasive examination of fetal cardiac function and has shown to be a sensitive method to detect subclinical cardiac condition and could be promising for assessment early fetal cardiac changes in normal and abnormal conditions. Our findings considered that maternal obesity alone may lead to initial subclinical alterations in fetal cardiac output. Also, it is an alarming finding in the existing high frequency of obesity nowadays and may partially clarify predilection of babies to cardiovascular disorder in advanced ages. A lot of investigations should focus on early fetal cardiac dysfunction, and the ability to detect early cardiac dysfunction may be valuable in monitoring and timing of delivery of complicated preterm pregnancy.

## Study limitation

There was a small number of patients and a lack of postnatal follow-up, and this is a single-center study.

## Data Availability

All data and equipment were available at the Tanta University Hospital.

## Abbreviations

BMI: Body mass index
TDI: Tissue Doppler imaging
2D-STE: Two-dimensional speckle-tracking echocardiography
STE: Speckle-tracking echocardiography
DM: Diabetes mellitus
FOW: Fetal of obese women
FDW: Fetuses of diabetic woman
FNW: Fetuses of normal pregnant woman
TDV: Tissue Doppler velocity
TAPSE: Tricuspid annular plane systolic excursions
GLS: Global longitudinal strain
SAPSE: Septal annular plane systolic excursion
MAPSE: Mitral annular plane systolic excursion
